# A Conjugate Based on Anti-HER2 Diaffibody and Auristatin E Targets HER2-Positive Cancer Cells

**DOI:** 10.3390/ijms18020401

**Published:** 2017-02-14

**Authors:** Anna M. Serwotka-Suszczak, Alicja M. Sochaj-Gregorczyk, Jerzy Pieczykolan, Daniel Krowarsch, Filip Jelen, Jacek Otlewski

**Affiliations:** 1 Department of Protein Engineering, Faculty of Biotechnology, University of Wroclaw, 50-137 Wroclaw, Poland; aniaserwotka@wp.pl (A.M.S.-S.); alicja.sochaj-gregorczyk@uj.edu.pl (A.M.S.-G.); 2 Preclinical Development Department, R&D Celon Pharma Inc., 05-092 Lomianki/Kielpin, Poland; jerzy.pieczykolan@celonpharma.com; 3 Department of Protein Biotechnology, Faculty of Biotechnology, University of Wroclaw, 50-137 Wroclaw, Poland; daniel.krowarsch@uwr.edu.pl; 4 Research and Development Department, Pure Biologics Ltd., 54-427 Wroclaw, Poland; filip@purebiologics.com

**Keywords:** targeted therapy, HER2, diaffibody, monomethyl auristatin E (MMAE)

## Abstract

Antibody-drug conjugates (ADCs) have recently emerged as efficient and selective cancer treatment therapeutics. Currently, alternative forms of drug carriers that can replace monoclonal antibodies are under intensive investigation. Here, a cytotoxic conjugate of an anti-HER2 (Human Epidermal Growth Factor Receptor 2) diaffibody with monomethyl-auristatin E (MMAE) is proposed as a potential anticancer therapeutic. The anti-HER2 diaffibody was based on the Z_HER2:4_ affibody amino acid sequence. The anti-HER2 diaffibody has been expressed as a His-tagged protein in *E. coli* and purified by Ni-nitrilotriacetyl (Ni-NTA) agarose chromatography. The molecule was properly folded, and the high affinity and specificity of its interaction with HER2 was confirmed by surface plasmon resonance (SPR) and flow cytometry, respectively. The (Z_HER2:4_)_2_DCS-MMAE conjugate was obtained by coupling the maleimide group linked with MMAE to cysteines, which were introduced in a drug conjugation sequence (DCS). Cytotoxicity of the conjugate was evaluated using the 3-(4,5-dimethyl-2-thiazolyl)-2,5-diphenyl-2-*H*-tetrazolium bromide MTT assay and the xCELLigence Real-Time Cell Analyzer. Our experiments demonstrated that the conjugate delivered auristatin E specifically to HER2-positive tumor cells, which finally led to their death. These results indicate that the cytotoxic diaffibody conjugate is a highly potent molecule for the treatment of various types of cancer overexpressing HER2 receptors.

## 1. Introduction

Antibody-drug conjugates (ADCs) are one of the most advanced approaches in the targeted treatment of cancers and are the leading cause of death in developed countries [1]. This promising strategy is based on the conjugation of monoclonal antibodies with cytotoxic drugs [2,3,4]. ADCs allow one to use the high selectivity of antibodies for the targeted delivery of cargo molecules directly to the tumor tissue [5,6]. Upon binding to tumor specific markers, ADCs undergo internalization followed by drug release in lysosomes, which eventually leads to cell death [7].

Currently, two ADCs, brentuximab vedotin (Adcetris, Seattle Genetics, Bothell, WA, USA) [8,9] and trastuzumab emtansine (Kadcyla, Genentech, South San Francisco, CA, USA) [10,11], are approved by the US Food and Drug Administration (FDA). A significant effort has been made to improve ADCs for the past 10 years and nowadays there are over 40 new ADC-based drugs under clinical trials [12]. The large size of monoclonal antibodies (MABs) (~150 kDa) imposes certain limitations, including slow blood clearance, high liver uptake, and poor tissue penetration [13,14]. To circumvent the limitations of antibodies, new drug carriers are being developed [15].

Alternative approaches were initially focused on antibody fragments, such as antigen-binding fragments (Fabs) [16,17], diabodies [18], single-chain variable fragments scFv [19], or nanobodies [20,21]. Recently, these constructs have been complemented with antibody mimetics, which rely on the proteins that specifically bind antigens but are not structurally related to antibodies [22]. Various protein scaffolds have been investigated, including trinectins [23], anticalins and lipocalins [24], designed ankyrin repeat proteins (DARPins) [25], and affibodies [26], for a wide range of biotechnological and therapeutic applications [27]. 

The affibody molecule was designed and developed by Affibody AB [28]. This 7 kDa protein is composed of 58 amino acids. The affibody molecule adopts a three-helix bundle structure that can be engineered to bind to a large number of target proteins or peptides with high affinity. It has a number of advantages over antibodies, including a smaller size and a simple, robust, tertiary structure, which results in lower production costs. With the use of phage display technology, affibodies for a wide range of targets can be readily developed [29]. 

The Z_HER2:4_ affibody specifically recognizes Human Epidermal Growth Factor Receptor 2 (HER2) [28]. HER2 plays an important role in cell growth, survival, and differentiation. It is also engages in major signaling pathways involving mitogen-activated protein kinase and phosphatidylinositol 3-kinase pathways [30]. HER2 gene amplification or HER2 protein overexpression contributes to the faster growth and spread of breast, ovarian, prostate, and several gastric cancers [31]. It has been estimated that HER2 overexpression occurs in 20%–30% of invasive breast cancers [32]. 

The small size of affibodies contributes to their short plasma circulation time and fast blood clearance, which makes them suitable for tumor imaging [33,34]. However, increasing the size of an affibody might be required to successfully use it as a drug carrier. Several attempts have been reported for extending the half-life of an affibody; for example, increasing the size of the protein by fusion with Albumin Binding Domain (ABD) [35]. 

A bivalent Z_HER2:4_ generated by Steffen et al. exhibited stronger binding between HER2 and (Z_HER2:4_)_2_, as well as increased internalization and cell retention compared to those of the monovalent affibody, and was evaluated in terms of tumor targeting in mice [36,37]. We took advantage of this dimeric format and constructed a diaffibody molecule containing a duplicated sequence of (Z_HER2:4_)_2_, with a drug conjugation site (DCS) at its C-terminus. The obtained construct is referred to as diaffibody (Z_HER2:4_)_2_DCS. The affibody molecule does not contain cysteines. Insertion of DCS, which has three cysteine residues, allowed us to couple a cytotoxic cargo via maleimide-thiol chemistry. Following purification from bacterial cells and biophysical analysis, (Z_HER2:4_)_2_DCS was conjugated with a highly potent drug called monomethyl auristatin E (MMAE), resulting in a potential anticancer biotherapeutic referred to as (Z_HER2:4_)_2_DCS-MMAE. The cytotoxic effect of our conjugate on HER2-positive cancer cells was confirmed in in vitro cytotoxicity assays, demonstrating that this cytotoxic conjugate, upon further in vivo evaluation, can serve as a potential anticancer agent. 

## 2. Results

### 2.1. Expression and Purification of the (Z_HER2:4_)_2_DCS Diaffibody

**Figure 1:**
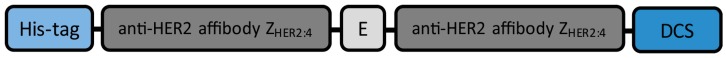
The (Z_HER2:4_)_2_DCS diaffibody construct is composed of two Z_HER2:4_ units separated by a single glutamate residue (E), a 6× His-tag at the N-terminus, and a drug conjugation sequence (DCS) at the C-terminus.

The diaffibody construct used in our study consists of a duplicated anti-HER2 affibody Z_HER2:4_ (Affibody AB) [28,36], separated by a single glutamate residue. The 6× His-tag was added at the N-terminus and a drug conjugation sequence (DCS) containing three cysteine residues (CAACAAAC) was added at the C-terminus of this construct (Figure 1). The diaffibody construct was cloned into the pET-30a vector (Novagen, Merck, Darmstadt, Germany). The protein was expressed in *E. coli* Bl21CodonPlus (DE3)-RIL cells and purified using the Ni-nitrilotriacetyl (Ni-NTA) agarose column. The final yield of the diaffibody was 20–25 mg from one litre of bacterial culture. The calculated molecular weight of the construct, 17,882 kDa, was confirmed by SDS-PAGE and mass spectrometry.

### 2.2. Structure and Thermal Stability of the (Z_HER2:4_)_2_DCS Diaffibody

**Figure 2:**
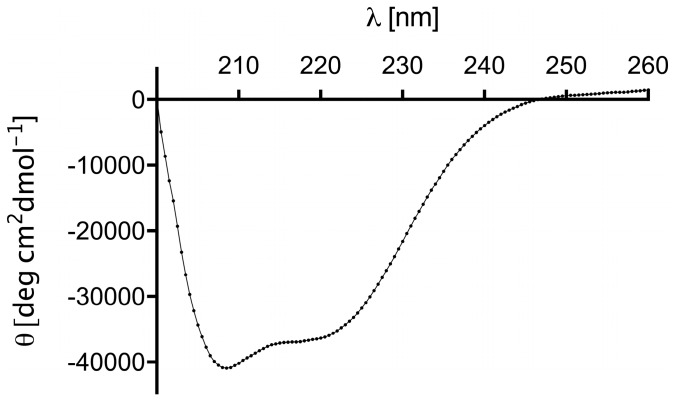
Circular dichroism (CD) spectrum of the diaffibody confirms a predominant α-helical secondary structure. Inset summarizes secondary structure content of (Z_HER2:4_)_2_DCS.

**Figure 3:**
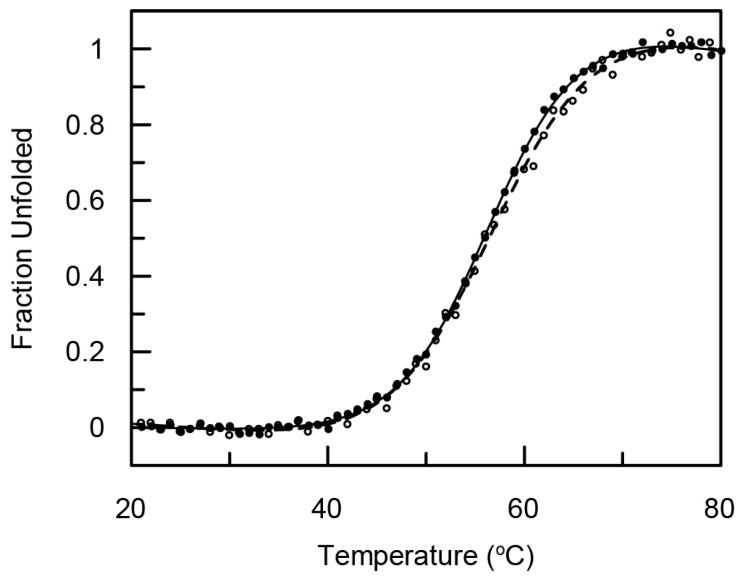
Normalized thermal denaturation (black line) and renaturation (dashed line) of (Z_HER2:4_)_2_DCS monitored by ellipticity changes.

The secondary structure of (Z_HER2:4_)_2_DCS was analyzed by circular dichroism (CD). The CD spectra were acquired in the range of 260 to 200 nm at 21 °C using 1 μM protein concentration and a 1 cm path length quartz cuvette. The CD spectrum was averaged over three scans (Figure 2). Analysis of the secondary structure content in the diaffibody showed that it represents a folded protein of α-helical structure. Quantitative analysis was performed using the DichoroWeb server, with the use of SELCON3 [38] and K2D algorithms, and CDpro software [39] using CDSSTR, SELCON3, and CONTIN/LL algorithms with SP43, SDP48, and SMP56 reference sets. Our results indicate that the (Z_HER2:4_)_2_DCS diaffibody contains more than 80% of α-helical structures. This is in accordance with the nuclear magnetic resonance (NMR) structure of a diaffibody protein that adopts a classical ‘up–down’ three-helical bundle fold [40]. To determine the stability of the designed protein, we performed thermal denaturation experiments (Figure 3). The denaturation process of (Z_HER2:4_)_2_DCS was monitored by circular dichroism (CD) in phosphate buffer, pH 7.4, at 222 nm. Thermodynamic parameters were calculated assuming a two-state reversible equilibrium transition. The denaturation temperature and van’t Hoff enthalpy are 57 °C and 46 kcal/mol, respectively. 

### 2.3. Specificity of the Dimeric Anti-HER2 Affibody

**Figure 4:**
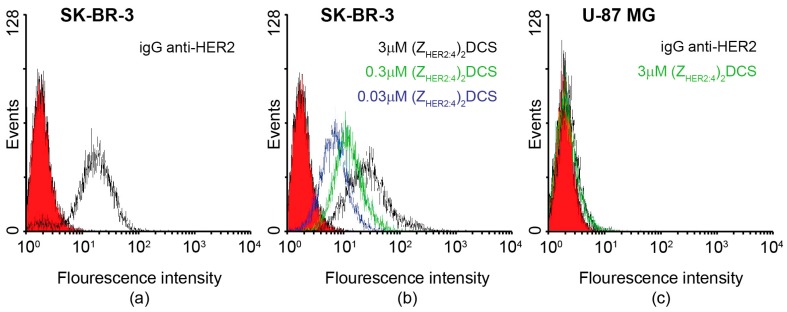
Specificity of the diaffibody-HER2 (Human Epidermal Growth Factor Receptor 2) binding analyzed by flow cytometry. (**a**,**b**) Positive staining was recorded for the HER2-positive SK-BR-3 cells with the anti-HER2 monoclonal antibody and with the fluorescently labeled diaffibody at three different concentrations: 0.03, 0.3 and 3 μM. (**c**) Banding is observed for the control HER2-negative U-87 MG cells.

In order to analyze by flow cytometry the specificity of the anti-HER2 diaffibody binding to HER2 present on cancer cells, (Z_HER2:4_)_2_DCS was fluorescently labeled with fluorescein isothiocyanate (FITC). Labeling was confirmed by mass spectrometry that showed traces of the unmodified diaffibody as well as the diaffibody labeled with one, two or three fluorescein molecules. The fluorescently labeled anti-HER2 diaffibody was used to stain the SK-BR-3 cells, which strongly overexpress HER2, and the control U-87 MG cells, which have physiological levels of HER2. The HER2 status of these cell lines was previously confirmed by SDS-PAGE analysis [41]. A similar experiment was also performed with commercially available anti-HER2 mouse monoclonal antibodies, followed by donkey anti-mouse polyclonal antibodies conjugated with FITC. Analysis of the histograms confirmed that diaffibodies bind to the HER2-positive cells in a concentration-dependent manner (Figure 4b) similar to the anti-HER2 monoclonal antibody (Figure 4a). As expected, the HER2-negative cells were not stained with either (Z_HER2:4_)_2_DCS-FITC or the anti-HER2 monoclonal antibody (Figure 4c).

### 2.4. vcMMAE Conjugation and Conjugate Characterization

#### 2.4.1. (Z_HER2:4_)_2_DCS-MMAE Preparation

**Figure 5:**
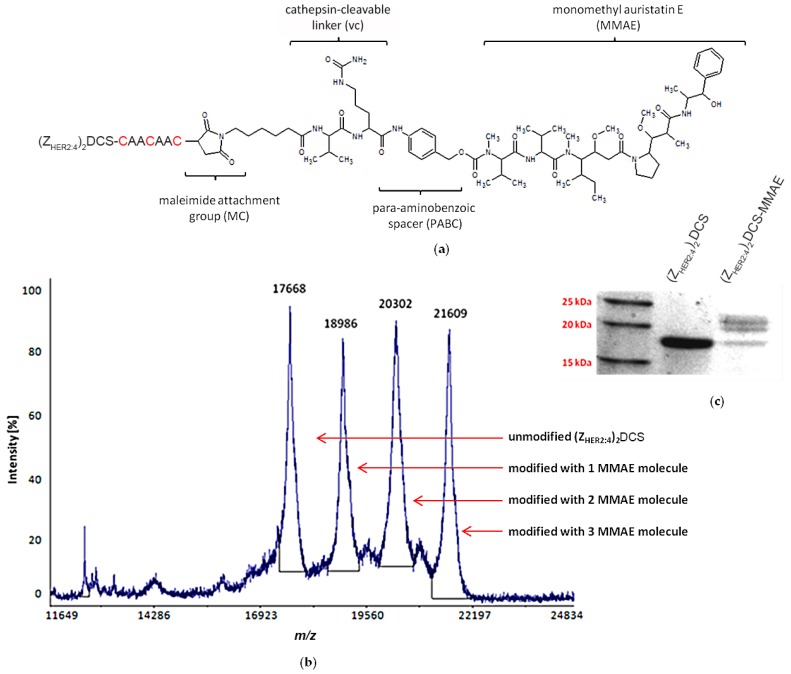
(Z_HER2:4_)_2_DCS conjugation with MC-Val-Cit-PABC-MMAE (monomethyl-auristatin E) (vcMMAE) (**a**) vcMMAE is attached to cysteine(s) present in the drug conjugation sequence via a valine-citrulline linker, which is cleaved by cathepsins inside tumor cells. The cleavage site is marked in red. (**b**) The mass spectrometry (MS) spectrum of the conjugation products showing the unmodified diaffibody and the diaffibody modified with 1, 2, and 3 vcMMAE molecules (**c**) SDS-PAGE separation of the conjugation mixture. Due to the low sensitivity of Coomassie brilliant blue staining in comparison to mass spectrometry, only two bands for the conjugate species were visualized.

MC-Val-Cit-PABC-MMAE (referred to as vcMMAE), which was used in this study, is composed of a maleimide attachment group (MC) that allows conjugation with the target protein via thiol groups, followed by a valine-citrulline (vc) linker and monomethyl auristatin E (MMAE). The linker is cleaved by cathepsins inside the endosomes of target cells. The MMAE molecule is separated from the cathepsin recognition site with a self-immolative p-aminobenzoic acid (PABC) spacer (Figure 5a). Prior to conjugation, (Z_HER2:4_)_2_DCS was reduced with 1 μM tris(2-carboxyethyl)phosphine (TCEP) for 1 h at room temperature. Following reduction, we used a 10-fold molar excess of vcMMAE and incubated the mixture for another 2 h. The conjugation occurred with about 70% efficiency. We attempted to separate the conjugation products using hydrophobic interaction chromatography (HIC), but we were unable to achieve a good separation. Therefore, the mixture was simply dialyzed against phosphate buffer saline (PBS). The mass spectrometry (MS) and SDS-PAGE analyses of the conjugation mixture showed that none, one, two, or three molecules of MMAE were conjugated to the diaffibody molecule due to the three cysteine residues inserted at the C-terminus of the diaffibody (Figure 5b,c). The conjugation product is referred to as (Z_HER2:4_)_2_DCS-MMAE.

#### 2.4.2. Analysis of (Z_HER2:4_)_2_DCS-MMAE Binding to HER2

**Figure 6:**
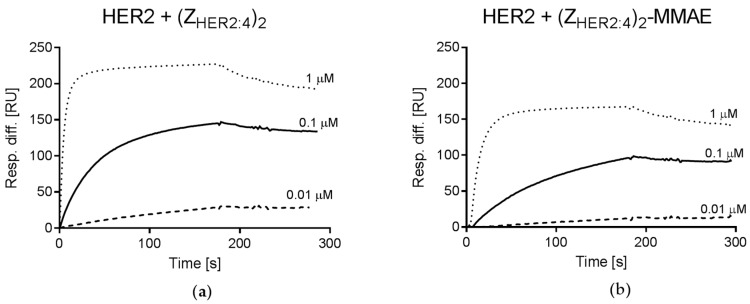
Surface plasmon resonance (SPR) analysis of HER2 and diaffibody interaction was performed using (**a**) increasing concentrations of 0.01, 0.1, and 1 μM of the diaffibody; and (**b**) the diaffibody-MMAE conjugate. The y axis represents the response difference in relative units (RU).

The affinity of the monomeric Z_HER2:4_ binding to HER2 was previously assessed for ~50 nM [28], while the dimeric construct bound HER2 with a higher affinity of ~3 nM [36]. To verify whether (Z_HER2:4_)_2_DCS retains the high affinity for HER2, we performed surface plasmon resonance (SPR) analysis. Recombinant HER2 protein was immobilized on the sensor chip and the diaffibody was injected at three concentrations; 0.01, 0.1, and 1 μM. The binding parameters were calculated using the simple 1:1 Langmuir binding model. The obtained data indicate that both (Z_HER2:4_)_2_DCS and (Z_HER2:4_)_2_DCS-MMAE bind to HER2 with high affinity, since the apparent dissociation equilibrium constant (K_D_) was about 18 nM for the diaffibody and for its conjugate (Figure 6). However, (Z_HER2:4_)_2_DCS and (Z_HER2:4_)_2_DCS-MMAE exhibited different association (k_on_) and dissociation (k_off_) rate constants. The k_on_ and k_off_ determined for (Z_HER2:4_)_2_DCS were 8.7 × 10^4^ M^−1^·s^−1^ (standard error (SE) = 1.3 × 10^4^) and 1.6 × 10^−3^ s^−1^ (SE = 1 × 10^−4^), respectively, whereas the conjugate was characterized by k_on_ of 6.4 × 10^4^ M^−1^·s^−1^ (SE = 3.5 × 10^2^) and k_off_ of 1.2 × 10^−3^ s^−1^ (SE = 4.5 × 10^−5^).

### 2.5. Cytotoxicity of the (Z_HER2:4_)_2_DCS-MMAE Conjugate

**Figure 7:**
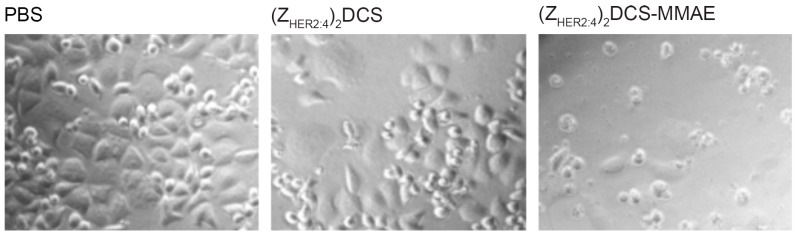
Microscopic analysis of SK-BR-3 cells treated with phosphate buffer saline (PBS), 500 nM (Z_HER2:4_)_2_DCS, and 500 nM (Z_HER2:4_)_2_DCS-MMAE after 72 h. The cells incubated with the anti-HER2 diaffibody did not show any morphological changes in comparison to the untreated control cells, whereas the viability of the cells treated with the conjugate was severely affected.

Prior to quantitative cytotoxicity assays on several cell lines, we decided to investigate whether the (Z_HER2:4_)_2_DCS-MMAE conjugate can affect the growth of HER2-positive (HER2+) breast cancer SK-BR-3 cells. The initial experiment relied on the microscopic observation of morphology and cell count of SK-BR-3 cells after 72 h incubation with 500 nM (Z_HER2:4_)_2_DCS-MMAE (Figure 7). 

**Figure 8:**
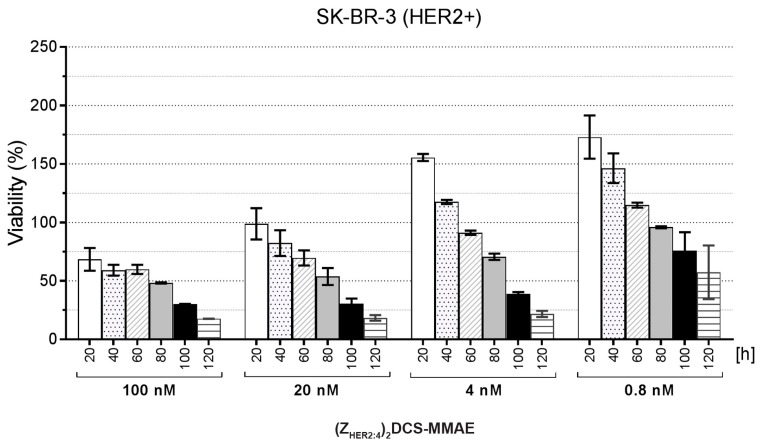
The xCELLigence experiment performed on SK-BR-3 cells treated with (Z_HER2:4_)_2_DCS-MMAE. Results are shown for the selected time points of 20, 40, 60, 80, 100, and 120 h. Results were normalized against the control cells treated with PBS at each time point. The error bars show the standard deviation.

The SK-BR-3 cells showed no morphological changes upon treatment with PBS and the unconjugated (Z_HER2:4_)_2_DCS molecule. In contrast, the cells treated for 72 h with 500 nM (Z_HER2:4_)_2_DCS-MMAE exhibited a round shape. We could also observe a significantly lower number of the cells that were attached to the culture plate surface, which indicated that the conjugate killed a vast majority of SK-BR-3 cells (Figure 7). Additionally, we monitored SK-BR-3 cells treated with different concentrations of our conjugate in real time using the xCELLigence Real-Time Cell Analyzer (ACEA Biosciences, San Diego, CA, USA) (Figure 8). The xCELLigence instrument measures change in electrical impedance, which depends on cell attachment to the culture plate with built-in microelectrodes. This allows for non-invasive, label-free cell death estimation. The obtained results confirmed that SK-BR-3 cells are sensitive to all the doses of (Z_HER2:4_)_2_DCS-MMAE used in this experiment. 

**Figure 9:**
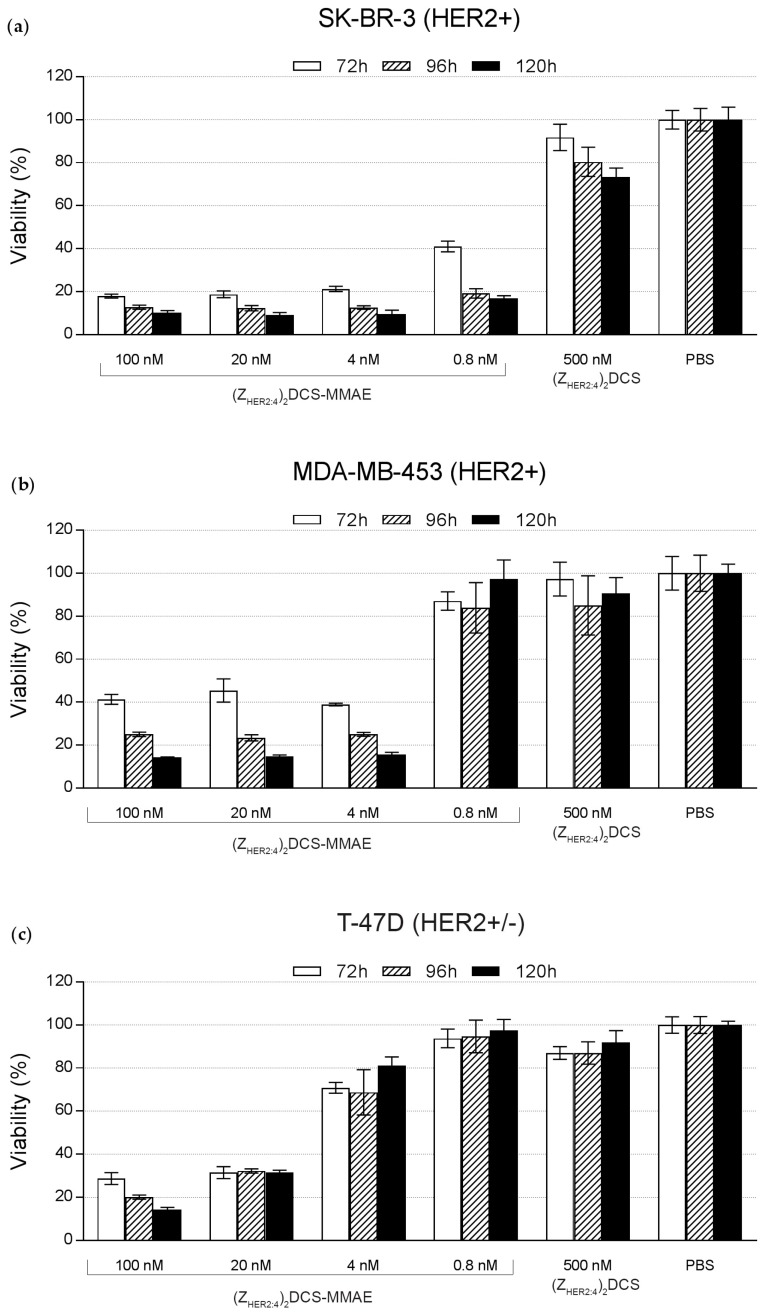
Viability assay results for human breast cancer cell lines (**a**) SK-BR-3 (HER2+); and (**b**) MDA-MB-453 (HER2+); and T-47D (HER2+/−) (**c**). These cells were incubated with the indicated doses of (Z_HER2:4_)_2_DCS-MMAE, 500 nM MMAE, and 500 nM (Z_HER2:4_)_2_DCS. Cell viability was assessed after 72, 96, and 120 h incubation. The error bars show the standard deviation.

To further evaluate the selectivity of (Z_HER2:4_)_2_DCS-MMAE in the colorimetric MTT assay, the following cell lines were used; SK-BR-3 and MDA-MB-453 HER2-positive (HER2+) cell lines; T-47D cells that exhibit a slight increase in HER2 levels (HER2+/−); and three HER2-negative cell lines (HER2−), U-87 MG, SK-MES-1, and MDA-MB-231 [41,42]. The above-mentioned cells were treated with increasing doses of the conjugate, and their viability was monitored after 72, 96, and 120 h. According to the obtained results, 0.8 nM (Z_HER2:4_)_2_DCS-MMAE reduced the SK-BR-3 cells’ viability to 15% within 120 h (Figure 9a). This concentration of the conjugate had a minor effect on the viability of MDA-MB-453 cells, which express lower levels of HER2 than SK-BR-3 cells [41,42], and no effect on the viability of T-47D cells (Figure 9). A 5-fold increase of (Z_HER2:4_)_2_DCS-MMAE concentration caused a decrease of MDA-MB-453 viability to 15% within 120 h (Figure 9b). To achieve a similar reduction of T-47D viability, we had to apply 100 nM (Z_HER2:4_)_2_DCS-MMAE for 120 h (Figure 9c). The half maximal inhibitory concentration (IC_50_) for SK-BB-3 cells treated with (Z_HER2:4_)_2_DCS-MMAE for 96 h was lower than 0.5 nM. This parameter was slightly elevated for MDA-MB-453 cells (IC_50_ = 1.9 nM) and T-47D cells (IC_50_ = 5.5 nM). The obtained results demonstrated that the effect of (Z_HER2:4_)_2_DCS-MMAE depends on the level of HER2 expression, since the (Z_HER2:4_)_2_DCS-MMAE potency increased along with the amount of HER2 expressed on the cell surface.

**Figure 10:**
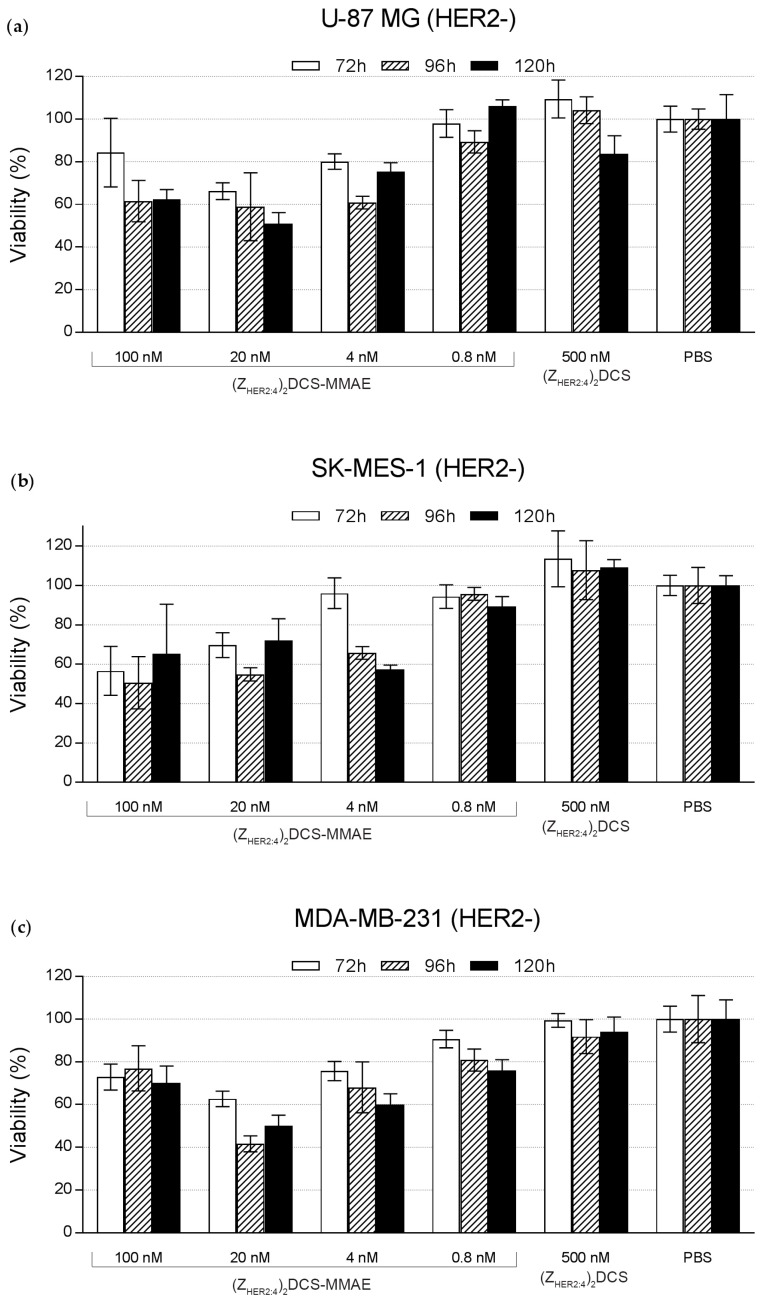
Viability assay results for the three HER2-negative cell lines (**a**) U-87 MG (human brain cancer); (**b**) SK-MES-1 (human lung cancer); and (**c**) MDA-MB-231 (human breast cancer). These cells were incubated with the indicated doses of (Z_HER2:4_)_2_DCS-MMAE, 500 nM MMAE, and 500 nM (Z_HER2:4_)_2_DCS. Cell viability was assessed after 72, 96, and 120 h incubation. The error bars show the standard deviation.

The MTT proliferation assay was also performed for the U-87 MG (Figure 10a), SK-MES-1 (Figure 10b), and MDA-MB-231 HER2-negative cell lines (Figure 10c). As expected, these HER2-negative cells were far less sensitive to (Z_HER2:4_)_2_DCS-MMAE than the HER2-positive cell lines, since the highest conjugate concentration (100 nM) reduced the cell viability to about 60%–80%. The IC_50_ values for U-87 MG, SK-MES-1 and MDA-MB-231 cell lines were above 100 nM.

## 3. Discussion

Recently, targeted anticancer therapies have become a powerful tool in medical practice due to increased efficacy and decreased side effects. Antibody-drug conjugates (ADCs) are effective in destroying cancer cells and are highly specific to selected targets. However, they are burdened with some disadvantages, including their large size that impairs tumor tissue penetration, a complicated structure that hampers their production in bacterial cells, and an extensive protection of intellectual property [13,14]. A promising approach to achieve a comparably selective treatment is targeted drug therapy based on the conjugates of an anticancer drug with non-antibody scaffolds [15,43].

Affibody is an example of a potential non-antibody drug carrier [41,44,45,46]. This small protein has been engineered to bind to a large number of target proteins or peptides with high affinity [29]. It is approximately 20 times smaller than antibodies and has a simple structure composed of three helices, which makes its production straightforward. Phage display has been used for the selection of affibodies interacting with particular molecular targets. Moreover, according to clinical studies, affibodies are non-immunogenic and well tolerated by patients [47,48]. One of the most commonly investigated affibodies is the Z_HER2:4_ affibody, which binds HER2. This protein kinase receptor is overexpressed in certain aggressive types of breast cancer and is one of the most common targets in cancer therapies [49,50].

We employed the (Z_HER2:4_)_2_DCS diaffibody, which is composed of two identical affibody sequences separated by a glutamic acid residue, the *N*-terminal His-tag, and the *C*-terminal drug conjugation sequence (DCS) as a drug-targeting molecule (Figure 1). Notably, a very similar construct, referred to as (Z_HER2:4_)_2_, was previously generated in order to improve the affinity and pharmacokinetics of the monovalent affibody [36,37]. (Z_HER2:4_)_2_DCS was overexpressed in the bacterial *E. coli* Bl21CodonPlus (DE3)-RIL strain and purified using an Ni-NTA column. The characterization of this protein by circular dichroism showed that the (Z_HER2:4_)_2_DCS diaffibody retains the α-helical structure of the parental protein (Figure 2). According to the denaturation experiments, the dimeric anti-HER2 affibody is a stable protein with a thermal denaturation temperature of 57 °C in a phosphate buffer (Figure 3).

The specificity of (Z_HER2:4_)_2_DCS binding to HER2 present on cancer cells was determined by flow cytometry (Figure 4). This method confirmed that (Z_HER2:4_)_2_DCS labeled with FITC specifically recognizes the SK-BR-3 HER2-positive cells. As expected, the HER2-negative U-87 MG cells were not detected by the construct. (Z_HER2:4_)_2_DCS was conjugated with monomethyl auristatin E (vcMMAE), resulting in the (Z_HER2:4_)_2_DCS-MMAE conjugate, which was confirmed by MS analysis and SDS-PAGE (Figure 5). The affinity of (Z_HER2:4_)_2_DCS and (Z_HER2:4_)_2_DCS-MMAE for HER2 was analyzed using surface plasmon resonance (SPR) (Figure 6a,b). According to our analysis, both (Z_HER2:4_)_2_DCS and (Z_HER2:4_)_2_DCS-MMAE bind HER2 with an apparent affinity of about 18 nM. This indicates that the auristatin conjugation to the *C*-terminal drug conjugation sequence does not influence the strength of diaffibody binding to HER2. However, the K_D_ obtained for our construct is six times higher than the K_D_ calculated for (Z_HER2:4_)_2_ (3 nM) [36]. This discrepancy may be caused by the presence of the drug conjugation sequence that enabled us to conjugate MMAE to (Z_HER2:4_)_2_. 

Our initial microscopic observation indicated that (Z_HER2:4_)_2_DCS-MMAE destroys SK-BR-3 cells that strongly overexpress HER2 on their surface (Figure 7). This result was also confirmed by the real-time cell viability analysis that demonstrated that the response of SK-BR-3 cells to (Z_HER2:4_)_2_DCS-MMAE is dose and time dependent (Figure 8).

To demonstrate that the conjugate can selectively target HER2 overexpressing cancer cells, we decided to use a broad spectrum of cell lines, including MDA-MB-453 cells expressing high levels of HER2 (HER2+), T-47D expressing slightly elevated levels of HER2 (HER2+/−), and cell lines expressing low levels of HER2 (HER2−), including U-87 MG, SK-MES-1, and MDA-MB-231. Importantly, we previously reported that all these cell lines are similarly sensitive to free MMAE [41,51]. The cytotoxicity of (Z_HER2:4_)_2_DCS-MMAE was measured by the MTT proliferation assay (Figure 9 and Figure 10). The MTT assay showed that the unconjugated (Z_HER2:4_)_2_DCS diaffibody had a minor negative effect on the viability of SK-BR-3 cells. This is consistent with the previous report by Ekerljung et al. that demonstrated that (Z_HER2:4_)_2_ inhibits proliferation of SK-BR-3 cells [52]. In the case of the SK-BR-3 and MDA-MB-453 cells that overexpress HER2, we observed a cytotoxic effect of the (Z_HER2:4_)_2_DCS-MMAE conjugate, causing a 90% decrease in cell growth after 120 h of the experiment in the concentration range from 100 nM to 4 nM (Figure 9). The IC_50_ values for the tested HER2-positive cells were within the low nanomolar range (from 0.5 nM for SK-BR-3 cells to 5.5 nM for T-47D cells). In contrast, the cytotoxicity of (Z_HER2:4_)_2_DCS-MMAE towards the HER2-negative cells was much lower than observed for the HER2-positive cells (Figure 10). This effect may be caused by a HER2-mediated endocytosis of the diaffibody-auristatin conjugate followed by auristatin release in endosomes, which leads to cell death. However, we cannot rule out the possibility that extracellular or cell surface associated proteases cleave the valine-citrulline linker and the released drug penetrates into cells. Therefore, the stability of the diaffibody-auristatin conjugate should be further addressed.

Importantly, the potency of the presented conjugate towards HER2-positive cells was comparable with ADC that has been successfully developed [53,54], and it can be further improved by obtaining homogeneous species of (Z_HER2:4_)_2_DCS loaded with three MMAE molecules. Overall, the cytotoxic conjugate based on the anti-HER2 diaffibody and MMAE efficiently destroys HER2-positive cancer cells in vitro. 

## 4. Materials and Methods

### 4.1. Cell Lines

The human adenocarcinoma cell line SK-BR-3 was maintained in McCoy’s 5A medium (Gibco, Thermo Fisher Scientific, Waltham, MA, USA) supplemented with 10% fetal bovine serum, 3 mM l-glutamine. and the appropriate antibiotics.The human glioblastoma epithelial cell line U-87 MG and the human lung squamous cell carcinoma derived from the metastatic site SK-MES-1 cell line were maintained in Minimum Essential Medium (MEM, Gibco) supplemented with 10% fetal bovine serum, 4 mM l-glutamine, and the appropriate antibiotics. The human ductal carcinoma from the mammary gland T-47D cell line, the human mammary gland MDA-MB-453 cell line, and the human mammary gland adenocarcinoma MDA-MB-231 cell line were maintained in Dulbecco’s Modified Eagle Medium (DMEM, Gibco) supplemented with 10% fetal bovine serum, 3 mM l-glutamine, and the appropriate antibiotics. All the cell lines were grown in a 5% CO_2_ atmosphere at 37 °C.

### 4.2. Protein Expression and Purification

The anti-HER2 diaffibody was designed on the basis of the anti-HER2 affibody Z_HER2:4_ sequence [28]. The amino acid sequence of Z_HER2:4_ affibody was duplicated in a head-to-tail configuration and separated with a glutamic acid residue linker. An *N*-terminal hexahistidyl (6× His) tag was added, allowing purification by immobilized metal ion affinity chromatography (IMAC), as well as the *C*-terminal drug conjugation site sequence (DCS), containing three cysteine residues (CAACAAAC). The constructed gene was cloned into the pET-30a vector (Novagen, Merck, Darmstadt, Germany). The protein was expressed in the *E. coli* Bl21CodonPlus (DE3)-RIL strain and purified using IMAC chromatography on Ni-NTA agarose (Qiagen, Hilden, Germany). The cell lysate was loaded onto an Ni-NTA agarose column equilibrated with 50 mM NaHPO_4_, 300 mM NaCl, and 10 mM imidazole. The column was then extensively washed with 50 mM NaHPO_4_, 300 mM NaCl, and 20 mM imidazole buffer, and the bound proteins were eluted with 50 mM NaH_2_PO_4_, 300 mM NaCl, and 250 mM imidazole buffer. Fractions containing diaffibody were pooled together and dialyzed overnight against PBS with 0.1 mM sucrose, 10% glycerol, and 0.1 mM TCEP. The purity of the diaffibody sample was analyzed by SDS-PAGE in reducing conditions. Protein bands were visualized by Instant Blue staining. Protein concentration was calculated from absorbance measurements at 280 nm, using the appropriate extinction coefficient (30,940 M^−1^·cm^−1^) and the molecular mass was verified by mass spectrometry analysis (MALDI TOF/TOF 4800, Applied Biosystems, Waltham, MA, USA).

### 4.3. Circular Dichroism

To verify the native conformation of the purified diaffibody, circular dichroism measurements were performed (J-715 spectropolarimeter, Jasco, Tokyo, Japan). Far-UV CD spectra in the range of 260–200 nm were acquired at 21 °C using 1 μM protein concentration at 68.5 mM NaCl, 1.35 mM KCl, 5 mM Na_2_HPO_4_, 0.9 mM KH_2_PO_4_, pH 7.4 buffer, and a 1 cm path length quartz cuvette. CD spectra were averaged over three scans and then converted to mean residue ellipticity [θ]. The secondary structure content was analyzed using tools available online; DichroWeb Server [55] with SELCON3 [35] and K2D algorithms and CDpro (Colorado State University, Fort Collins, CO, USA) software [36] using CDSSTR, SELCON3, and CONTIN/LL algorithms with SP43, SDP48, and SMP56 reference sets. Thermal denaturation experiments were conducted by monitoring changes in the CD signal at 222 nm between 20 °C and 80 °C at a constant rate of 1 °C/min during the denaturation and renaturation processes. Denaturation data were analyzed using PeakFit software (Systat Software, San Jose, CA, USA), assuming a two-state reversible equilibrium transition as described previously [56].

### 4.4. Affinity Measurements

The recombinant extracellular domain of HER2 (Sino Biological, Beijing, China) was diluted in acetate buffer with pH 6.0 and immobilized (1000 resonance units (RU)) onto the surface of a CM5 sensor chip by standard amine coupling. The binding analysis was carried out with 0.01, 0.1, and 1 μM (Z_HER2:4_)_2_DCS and (Z_HER2:4_)_2_DCS-MMAE diluted in 10 mM HEPES, 150 mM NaCl, 3 mM EDTA, and 0.02% sodium azide, with pH 7.4, using a BIAcore 3000 instrument (GE Healthcare, Chicago, IL, USA). The running conditions were 10 μL/min flow rate, 25 °C, a 3 min association time, and a 2 min dissociation time. Following dissociation, the chip was regenerated with 50 mM NaOH as the regeneration buffer. All the buffers were filtered and degassed prior to each experiment. The dissociation equilibrium constant (K_D_) was determined using BIA evaluation 3.2 software (Biacore, GE Healthcare, Chicago, IL, USA), assuming one-to-one binding.

### 4.5. Fluorescein Labeling

Prior to coupling to 5-iodoacetamidofluorescein (5-IAF) (Thermo Fisher Scientific, Waltham, MA, USA), (Z_HER2:4_)_2_DCS was reduced with 1 μM TCEP for 1 h at room temperature. 5-IAF was added to a final concentration of 10 times molar excess over each of three cysteines in the protein. The sample was incubated for 2 h with shaking. After incubation, the sample was dialyzed against PBS overnight. The efficiency of the reaction was confirmed by mass spectrometry analysis. The conjugation product is referred to as (Z_HER2:4_)_2_DCS-FITC.

### 4.6. Flow Cytometry

The U-87 MG and SK-BR-3 cells were trypsinized, washed three times with PBS, and incubated for 1 h with (Z_HER2:4_)_2_DCS-FITC at the concentrations of 0.03, 0.3, and 3.0 μM. As a control, the cells were incubated for 1 h with mouse monoclonal anti HER-2 antibody (AbD Serotec, Bio-Rad, Hercules, CA, USA), washed three times with PBS, and again incubated for 1 h with FITC-conjugated donkey anti-mouse antibody (Jackson Immuno Research, West Grove, PA, USA). Cells were washed three times with PBS and fixed with 1% paraformaldehyde, following their analysis on a FACSDiva instrument (BD Biosciences, Franklin Lakes, NJ, USA). The obtained results were analyzed using WinMidi software (Purdue University, West Lafayette, IN, USA).

### 4.7. vcMMAE Conjugation

Prior to conjugation to MC-Val-Cit-PABC-MMAE (Chiro Block, Wolfen, Germany), (Z_HER2:4_)_2_DCS was reduced with 1 μM TCEP for 1 h at room temperature. Following reduction, MC-Val-Cit-PABC-MMAE was added to a final concentration of 10 times molar excess over the protein. The sample was incubated for 2 h with shaking. After incubation, the sample was dialyzed against PBS and the product of the reaction was analyzed by mass spectrometry.

### 4.8. Mass Spectrometry

MALDI-TOF MS was performed in positive ion mode. Protein samples (1–2 µL) were spotted onto the metal plate of an MALDI TOF/TOF 4800 (Applied Biosystems, Waltham, MA, USA) in serial dilutions with 0.1% trifluoroacetic acid (TFA) in 50% (*v*/*v*) acetonitrile. After drying, 1 µL of α-Cyano-4-hydroxycinnamic acid (CHCA) (Sigma-Aldrich, St. Louis, MO, USA) solution (10 mg/mL) was freshly dissolved in 50% (*v*/*v*) acetonitrile containing 0.1% TFA. After drying, the spectra were recorded within the 200–20,000 Da range. For each spot, spectra were obtained from 1000 laser shots (40 subspectra, 25 shots/subspectrum) with a 200 s^−1^ laser shot frequency and a laser power of 3500–4700 AU. Protein samples in complex buffers were extracted with ZipTip 18C (Millipore, Billerica, MA, USA).

### 4.9. Cytotoxicity Measurements

The cytotoxicity of the (Z_HER2:4_)_2_DCS-MMAE conjugate was measured using the MTT proliferation assay and xCELLigence impedance-based, label-free, real-time cell analyzer (ACEA Biosciences, San Diego, CA, USA). For the MTT assay, cells were seeded on 96-well plates at 7500 cells per well in 100 μL of medium and were cultured for 24 h. (Z_HER2:4_)_2_DCS-MMAE at final concentrations of 100, 20, 4, and 0.8 nM was added to the cell cultures and incubated for 72, 96, and 120 h. After incubation, 20 μL of the MTT reagent was added to each well and the plates were incubated at 37 °C. Following 4 h incubation, the cells were lysed by the addition of 80 μL of lysis buffer (45% dimethylformamide (DMF), 13.5% sodium dodecyl sulfate (SDS)). The fluorescence was measured at 590 nm with a reference filter of 620 nm using the Envision Multimode Plate Reader (PerkinElmer, Waltham, MA, USA). For the xCELLigence assay, the cells were seeded in plates at 10,000 cells per well in the appropriate medium and cultured for 24 h in standard conditions. The (Z_HER2:4_)_2_DCS-MMAE conjugate at final concentrations of 100, 20, 4, and 0.8 nM was added to the cell cultures and cell viability was monitored for 120 h with 1 h intervals. Cells were also analyzed using a light microscope.
