# Correction: Serwotka-Suszczak, A. M. et al. A Conjugate Based on Anti-HER2 Diaffibody and Auristatin E Targets HER2-Positive Cancer Cells. *Int. J. Mol. Sci.* 2017, *18*, 401

**DOI:** 10.3390/ijms19113676

**Published:** 2018-11-20

**Authors:** Anna M. Serwotka-Suszczak, Alicja M. Sochaj-Gregorczyk, Jerzy Pieczykolan, Daniel Krowarsch, Filip Jelen, Jacek Otlewski

**Affiliations:** 1 Department of Protein Engineering, Faculty of Biotechnology, University of Wroclaw, 50-137 Wroclaw, Poland; aniaserwotka@wp.pl (A.M.S.-S.); alicja.sochaj-gregorczyk@uj.edu.pl (A.M.S.-G.); 2 Drug Discovery Department, Adamed Group, 05-152 Czosnow, Poland; jerzy.pieczykolan@celonpharma.com; 3 Department of Protein Biotechnology, Faculty of Biotechnology, University of Wroclaw, 50-137 Wroclaw, Poland; daniel.krowarsch@uwr.edu.pl; 4 Research and Development Department, Pure Biologics Ltd., 54-427 Wroclaw, Poland; filip@purebiologics.com

It has been brought to our attention that the affiliation of Dr. Jerzy Pieczykolan at the time when he was responsible for the work described in the paper [1] was not mentioned in the previous published version.

We would like to change the Dr. Jerzy Pieczykolan’ affiliation on Page 1 of paper [1] from:

Preclinical Development Department, R&D Celon Pharma Inc., 05-092 Lomianki/Kielpin, Poland; jerzy.pieczykolan@celonpharma.com.

to the correct version, as follows:

Drug Discovery Department, Adamed Group, Czosnow 05-152, Poland; jerzy.pieczykolan@adamed.com.pl.

And add the following affiliation as the current address of Dr. Jerzy Pieczykolan:

Preclinical Development Department, R&D Celon Pharma Inc., 05-092 Lomianki/Kielpin, Poland; jerzy.pieczykolan@celonpharma.com.

This correction does not cause any changes to results and conclusions in the original published paper.
